# Influenza Vaccination Strategies: Comparing Inactivated and Live Attenuated Influenza Vaccines

**DOI:** 10.3390/vaccines3020373

**Published:** 2015-04-24

**Authors:** Saranya Sridhar, Karl A. Brokstad, Rebecca J. Cox

**Affiliations:** 1 Jenner Institute, University of Oxford, Oxford OX3 7DQ, UK; 2 Broeglemann Research Laboratory, Department of Clinical Science, University of Bergen, N-5021 Bergen, Norway; E-Mail: Karl.brokstad@k2.uib.no; 3 Influenza Centre, Department of Clinical Science, University of Bergen, N-5021 Bergen, Norway; E-Mail: rebecca.cox@k2.uib.no; 4 Department of Research and Development, Haukeland University Hospital, N-5021 Bergen, Norway; 5 Jebsen Centre for Influenza Vaccine Research, University of Bergen, N-5021 Bergen, Norway

**Keywords:** Influenza, vaccination, immunization, live attenuated vaccine, immunity, antibodies, T-cells

## Abstract

Influenza is a major respiratory pathogen causing annual outbreaks and occasional pandemics. Influenza vaccination is the major method of prophylaxis. Currently annual influenza vaccination is recommended for groups at high risk of complications from influenza infection such as pregnant women, young children, people with underlying disease and the elderly, along with occupational groups such a healthcare workers and farm workers. There are two main types of vaccines available: the parenteral inactivated influenza vaccine and the intranasal live attenuated influenza vaccine. The inactivated vaccines are licensed from 6 months of age and have been used for more than 50 years with a good safety profile. Inactivated vaccines are standardized according to the presence of the viral major surface glycoprotein hemagglutinin and protection is mediated by the induction of vaccine strain specific antibody responses. In contrast, the live attenuated vaccines are licensed in Europe for children from 2–17 years of age and provide a multifaceted immune response with local and systemic antibody and T cell responses but with no clear correlate of protection. Here we discuss the immunological immune responses elicited by the two vaccines and discuss future work to better define correlates of protection.

## 1. Introduction

Influenza continues to remain among the most important respiratory infections causing annual seasonal epidemics and the occasional pandemic. Influenza virus causes an estimated annual global toll of 500,000 deaths and 1 billion severe illnesses and an estimated $ 8 billion in annual economic cost of influenza in the US alone [1].

Among the various public health strategies in place to combat influenza, vaccination is the most cost-effective strategy against annual seasonal influenza. Inactivated “killed” influenza vaccines have been in use since the 1940s with improvements primarily made in production technologies and use of adjuvants. An alternative type, live attenuated influenza vaccine, has been in use in Russia for over 50 years and in 2003 was licensed for use in North America. More recently, Europe has licensed this vaccine and recommended its use in children from 2–18 years of age. Although this new live attenuated influenza vaccine (LAIV) has been in development since the 1970s and extensive data on safety and efficacy is available, the immunological mechanisms of action and correlates of protection remain unclear. Here we review our current understanding of the efficacy of LAIV in humans, compare trivalent inactivated influenza vaccine (TIV) to LAIV and highlight the key research questions that will impact immunization policies with LAIV.

## 2. Epidemiology of Influenza

There are three types of influenza virus (A, B and C), which differ in their epidemiology, pathogenicity, antigenicity and genome organization. Type A is the most common type found in a wide variety of birds and mammals, while types B and C are predominantly human pathogens. Influenza A virus is further subdivided into different subtypes based on antigenic differences in the surface glycoproteins, hemagglutinin (HA) and neuraminidase (NA). Annual seasonal influenza epidemics are caused by subtypes of Influenza A and Influenza B viruses while Influenza A viruses are responsible for influenza pandemics.

**Figure 1:**
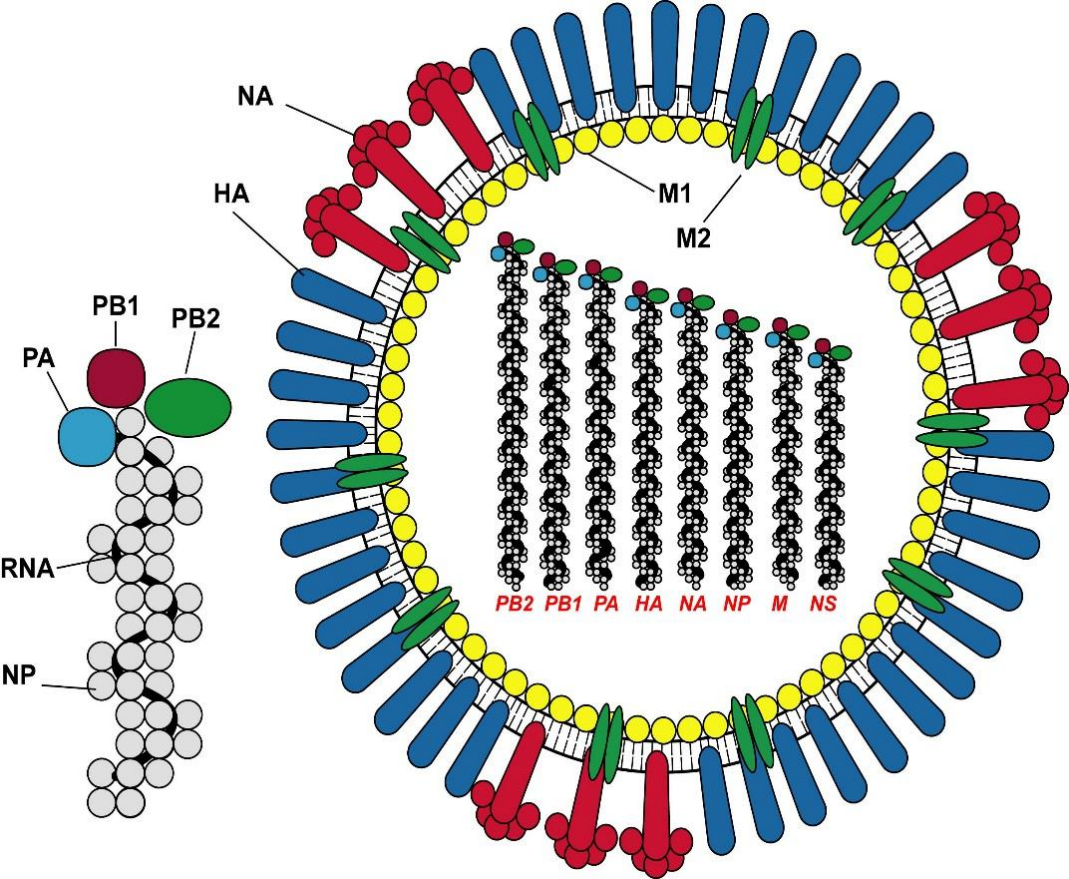
The structure of Influenza A virus and the ribonucleoprotein complex. The virus proteins are denoted as HA hemagglutinin; NA neuraminidase, M1 matrix protein 1; M2 matrix protein 2; NP nucleoprotein; and the polymerase proteins PA, PB1 and PB2.

Influenza is an enveloped negative sense segmented RNA virus with two surface glycoproteins and nine internal proteins (Figure 1). The surface glycoproteins, HA and NA enable attachment, entry and egress of the virus from infected cells. The HA trimer protein has a globular head region with sialic acid receptor binding sites that enable attachment to host cells. Mutations in these receptor-binding sites on the globular head are responsible for the antigenic variation that generates drift variant virus strains responsible for seasonal outbreaks of influenza. At unpredictable intervals, different subtypes of influenza A virus undergo gene reassortments to give rise to a novel virus strain that is capable of causing pandemics in the immunologically naïve population. Once a pandemic virus emerges, it usually replaces the previously circulating Influenza A strain of the same subtype, as seen with the 2009 H1N1pdm09 virus. In addition, avian influenza viruses, e.g., H5N1 or H7N9 can cause human infection following close contact with infected poultry but to date remain non-transmissible between humans.

Children and the elderly are at particularly high risk of developing severe disease following influenza infection. Severe disease with influenza is usually manifest by respiratory failure, acute respiratory distress syndrome (ARDS) and secondary bacterial pneumonia. In addition to these traditional high-risk groups, asthmatics, those with chronic lung disease, those with liver disease, immunosuppressed individuals, pregnant women and diabetics are also considered high-risk target groups. These groups are targeted for immunization by annual vaccination programs.

## 3. Vaccine Design and Recommendations

Seasonal influenza vaccines are composed of virus strains representative or antigenically similar to those circulating in the population for that season. Traditionally influenza vaccines have been trivalent to cover Influenza A H3N2, H1N1 and influenza B strain. The circulation of two lineages of influenza B has led to development and licensing of quadrivalent vaccines containing both influenza B lineages. However, in order to achieve this, experts need to predict the circulating strain in order to make the vaccine for the upcoming season, in part because influenza vaccines are predominantly produced in embryonated hen’s eggs which takes approximately 6 months. In February every year an expert panel that includes the World Health Organization (WHO) reviews Southern hemisphere data and decides on the circulating strains for the subsequent influenza season in the Northern hemisphere. Manufacturing of vaccines is initiated in March incorporating the strains recommended by the WHO. A major limitation of this approach is the dependence on the influenza viruses not undergoing antigenic change (drift or shift). Ever so often, new antigenic drift variants emerge to which the vaccines are poorly matched such as the influenza A H3N2 in 2014–2015 season in the Northern hemisphere. In such cases, vaccine efficacy has been shown to be 50% lower depending on the degree of the match [2]. Moreover, if pandemic strains emerge, manufacture is not rapid enough to produce vaccines against these new strains. Thus, there have been significant efforts into developing better ways to predict antigenic change in influenza viruses, developing new techniques to reduce manufacturing time, developing universal vaccines that are effective against a broad range of viruses and developing vaccine stockpiles.

**Figure 2:**
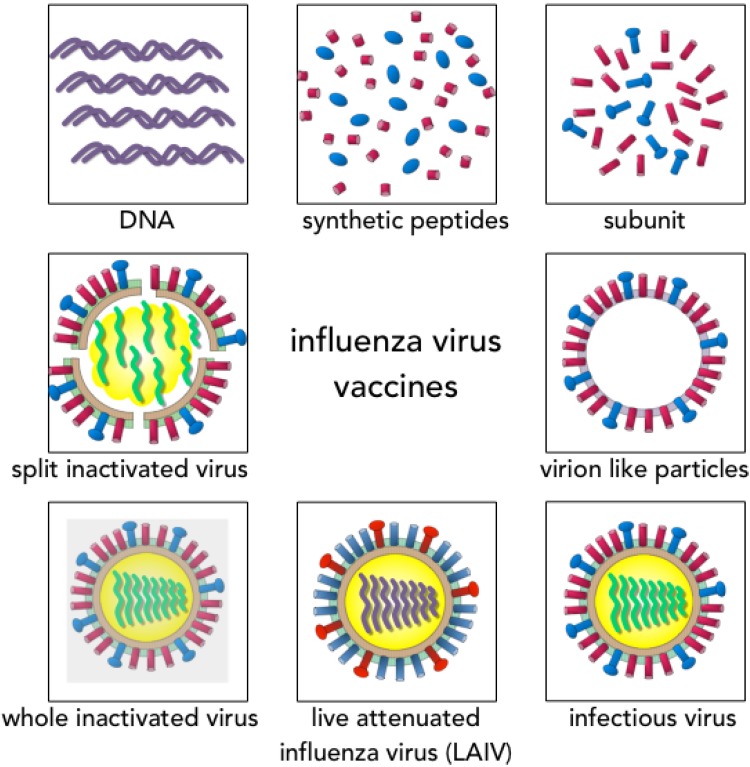
The different formulations of influenza vaccine. Currently licensed influenza vaccines are predominately inactivated virus (whole inactivated, split, subunit or virion like particle) or live attenuated influenza vaccine. Novel vaccines are DNA or synthetic peptide vaccines.

Annual influenza vaccination recommended for target groups are either inactivated vaccines or, more recently, live attenuated vaccines (Figure 2). Inactivated vaccines are either split virus, subunit vaccines or recombinant HA based vaccines that are administered parenterally. The vaccines are standardized according to the quantity of hemagglutinin, commonly 15 μg HA per strain, although high dose vaccines have recently been licensed for the elderly containing 60 μg HA per strain [3]. Adjuvants like oil in water (MF5 and AS03) increase the immunogenicity of the vaccine and are particularly used in the elderly, and for pandemic vaccines to spare doses [4]. Inactivated vaccines have an excellent safety profile, are recommended for children from 6 months of age, the elderly, asthmatics and those with other high risk conditions (Table 1).

**Table 1:** The World Health Organization Strategic advisory committee influenza vaccine recommendations in prioritized order.

Recommended Group	WHO Rationale for the Recommendation
Pregnant women	Increased risk of serious disease in mother
	Increased risk of death in mother and unborn child
	Secondary effect of protection of child up to 6 m
	Globally applicable *
Healthcare workers	Increased exposure to influenza
	Reduces morbidity and mortality in patients
	Preserves integrity of health care systems
	Possible to implement
Children <2 years old	Experience highest levels of serious illness
	Responsible for spread in community
	Disadvantage costly to implement vaccination campaign
Children 2–5 years old	Large burden of morbidity
	Respond better to vaccines than younger children
	live attenuated influenza virus (LAIV) gives improved protection
Children < 6 months	No available vaccines
	Indirect protection through vaccination of mother during pregnancy
	Indirect protection through vaccination of close contacts
Elderly > 65 years old	Highest risk of mortality
	Vaccine is less effective
	Disadvantage annual immunization is costly to administer
Patients with chronic conditions	Highest risk for serious disease
	Disadvantage requires considerable resources to identify individuals

* pregnant women have contact with health care services.

In contrast, intranasally administered live attenuated influenza vaccines are produced by reverse genetics using the HA and NA genes from circulating viruses on an attenuated, temperature-sensitive, cold adapted virus backbone. This backbone prevents replication at temperatures above 33 °C, thereby restricting replication to the upper but not lower respiratory tract [5,6]. Although the master donor backbone varies between the different manufacturers of the LAIV vaccine, the safety profile of this vaccine in immunocompetent adults and children is good, and multiple studies have demonstrated the genetic stability of the live vaccine virus strains [7]. Once administered, vaccine virus can be isolated from nasal secretions up to 7 days post-vaccination in young children, but is rarely observed for longer than 14 days post-vaccination, and secondary transmission of the virus to close contacts is uncommon [8]. In the US and Canada, this vaccine is recommended for children from 2 years of age and for healthy adults up to 49 and 59 years of age, respectively. In Europe, the recommendation is limited to children 2–18 years of age [9]. In immunologically naïve subjects (e.g., children <9 years old, who have not been previously vaccinated with influenza) two doses of vaccine are recommended at minimum of 4-week interval. In children 2–8 years old who have previously been vaccinated or children (>8 years old) and adults, one dose of vaccine is recommended. The LAIV is preferentially recommended for vaccination of children in some countries such as the UK where it is included in the childhood vaccination program and in Germany [10], although it was recently removed from preferential recommendation in the USA after poor efficacy had been observed against the Influenza A H1N1 and drifted H3N2 strains [11]. However, this live virus vaccine is not recommended for children under 2 years of age, in the elderly, those immunosuppressed and those caring for people with high risk of severe influenza disease. Also, LAIV is contraindicated in severe asthmatics currently on oral or high dose inhaled glucocorticosteriods or active wheezing. In early studies, when the live virus was administered in children under 2 years of age, an increase in wheezing was observed in vaccinated children, which underlies the rationale for not recommending the vaccine for under 2-year-olds [12] (Table 2).

**Table 2:** LAIV is contraindicated in the following people.

Children	General Contradictions in all Groups
< 24 months of age Receiving aspirin or aspirin-containing therapy ^1^	Hypersensitivity to gelatin, gentamicin or ovalbumin Pregnant women in USA and Canada Older adults (USA >50 & Canada >60 years old)
	Clinical immunodeficiency due to conditions or immunosuppressive therapy ^2^

^1^ Association of Reye’s syndrome with salicylates and wild-type influenza infection; ^2^ acute and chronic leukemias; lymphoma; symptomatic HIV infection; cellular immune deficiencies; and high-dose corticosteroids.

## 4. Natural Immunity To Influenza

Exposure with influenza virus initiates a cascade of humoral and cellular immune responses to resist infection and development of symptoms. Understanding these protective immune responses and the mechanisms by which they are induced are critical for the development of new improved vaccines that attempt to induce such protective responses.

The primary mediator of protection against infection is neutralizing antibodies targeting the HA. These antibodies are predominantly targeted against the receptor-binding sites in the distal globular head region of the virus thereby preventing attachment of the virus to host cells. The measurement of HA specific antibodies by the Hemagglutination-Inhibition (HAI) and Microneutralization (MN) assays remains the primary correlate of protection against influenza and current influenza vaccines are designed to induce such strain-specific antibodies [13]. In contrast to such strain-specific antibodies, antibodies targeting the highly conserved membrane proximal stalk region of HA have been recently shown to mediate protection against a broad range of viruses [14,15]. This broad cross-strain protection is particularly important as new virus strains emerge to which host neutralizing antibodies are less effective. Although the exact mechanisms by which these stalk-specific antibodies mediate protection is unclear, prevention of fusion, preventing release of viral progeny and antibody-dependent cellular cytotoxicity (ADCC) may account for their protective effect. Antibodies to other viral proteins such as NA and M2e are also involved in mediating protection although their exact contribution remains unclear. Anti-NA antibodies have been shown to protect against development of illness following natural infection and following experimental challenge [16,17]. However, the assays to measure these antibodies are technically difficult to standardize and work is ongoing to standardize assays to measure these antibodies. Anti-M2e antibodies constitute a particularly attractive target for vaccine development, as M2e is a highly conserved protein on the viral surface to which antibodies can be targeted. In animal models, antibodies to M2e have been shown to mediate protection against lethal influenza [18], although the role for these antibodies in protection in humans remains to be confirmed.

In contrast to antibodies, T-cells can protect against development of symptomatic disease when antibody mediated protection against infection is circumvented. The role of T-cells in mediating protection has been extensively demonstrated in animal and non-human primate models [19,20]. In experimental challenge models in humans, pre-existing CD4+ and CD8+ T-cells were shown to be associated with reduced viral shedding and symptoms [21,22]. More recently, we have identified CD8+ T-cells of a late-effector (CD45RA^+^CCR7^−^) phenotype to be associated with protection against symptomatic influenza [23]. Critically, these T-cells are capable of recognizing epitopes on internal proteins that are highly conserved across different influenza viruses. The role of CD4+ T-cells in mediating protection against influenza is becoming increasingly evident. CD4+ T-cells are capable of direct cytotoxic killing of virus-infected cells as well as providing help to CD8+ T-cells [24]. A new subset of CD4+ T-cells, T follicular helper cells (T_FH_) have been described which are vital in B cell help, germinal center formation and induction of antibodies after infection and vaccination [25].

A key requirement for mediating protection against influenza is the necessity for immune mediators to be present at the site of infection—the respiratory tract. Mucosal IgA antibodies induced following infection and intranasal vaccination correlate with protection against infection in experimental human challenge models of influenza [26]. Mucosal T-cells mediate protection against influenza in animal models, although their role in humans is less clear. Mucosal T-cells include T-cells in the airway, lung-migrating memory T-cells and lung-resident memory T-cells have been demonstrated to mediate protection in animal models of influenza [27,28,29].

Natural infection is able to induce this multifaceted mucosal and systemic immune response to confer protection against subsequent influenza infection with similar strains. Thus, the challenge for an effective influenza vaccine is to mimic natural infection in conferring protection against influenza.

## 5. Inactivated Influenza Vaccines (IIV)

Inactivated influenza vaccines (IIV) have been in production and in use since the 1940s and are the most common type of influenza vaccines produced and used. These vaccines primarily contain HA and NA proteins with some preparations containing some NP protein with or without an accompanying adjuvant. Meta-analyses have found inactivated vaccines to show ~60% efficacy in children and ~40% efficacy in adults and the elderly [2,31], although a more recent meta-analysis with stringent inclusion criteria for trials found lower levels of efficacy than previously reported [32].

Previously the European Medicines Agency (EMA) committee for Medicinal Products for Human use (CHMP) required vaccine manufacturers to conduct clinical trials each year to examine the tolerability and immunogenicity measured by the HAI assay for annual updating of seasonal IIV [33]. In 2015 these guidelines were changed to no longer require clinical trials for strain changes of licensed IIV [34]. The evidence for use of HAI titers as a surrogate correlate of protection comes from human challenge and infection studies. In human challenge studies, pre challenge serum HAI titer of 18–36 was associated with 50% protection from infection [35]. A clear relationship between HAI titers prior to infection and the percentage of people infected was found in subsequent studies with viral [36] and attenuated viral challenge [37] and in a recent meta-analysis [38]. These studies support the use of evaluation of serum HAI antibody responses as a surrogate correlate of protection in adults for investigating vaccine responses. However in children despite a correlation between the presence of HAI antibody and protection from infection with the same strain [39], higher HAI titers of 110 were required for 50% protection [40]. The HAI assay is a technically simple assay to perform relying on the ability of HA specific antibodies to inhibit the binding of HA to red blood cells, however international standardization studies have shown that there is large variation in HAI titers (8–128-fold) between laboratories [41,42,43,44]. Therefore, there is a need to define the HAI titers associated with laboratory confirmed influenza (virus culture or PCR positive) in all populations which could be used in evaluation of vaccines responses and importantly to define HAI titers associated with protection to pandemic strains.

Vaccination with IIV results in both local and systemic immune responses. The serum antibody response increases as early as 2–6 days after seasonal inactivated influenza vaccination in primed subjects [45] and peaks at approximately 2 weeks after vaccination when 90% of vaccinees have protective antibody titers [45,46] The durability of antibody responses following IIV is not clear. The serum antibody response then wanes over time and is generally two-fold lower 6 months after vaccination [47], although absolute titers seem to be maintained above the protective threshold. A seminal study investigating the durability of protection after vaccination and correlating this with antibody responses found that although there was a decline in the antibody responses within 8 months after IIV, although individuals were still protected against experimental challenge from a live attenuated homologous virus [48].

The serum antibody response is dominated by the IgG, particularly IgG1, isotype of antibodies with lower concentrations of IgM and IgA. Influenza-specific antibody-secreting cells (ASC) appear in the circulation at approximately 7 days post seasonal influenza vaccination, earlier than serum antibody response [45] and consist predominantly of IgG and IgA. In young children (2 to 3 years old), previous natural priming by influenza infection was essential to mount strong antibody and antibody secreting cell responses in the peripheral blood [49,50] after inactivated vaccine. In these children, peak ASC response was observed 7 days after the first dose and 4–6 days after the second vaccine dose. In healthy adults, high numbers of influenza specific antibody secreting cells (ASC) are present in the nasal mucosa but the numbers remain stable after inactivated vaccination [51]. However, a rapid transient increase in specific ASC is observed in the tonsils and peripheral blood after parenteral vaccination [45,52]. Generally peak ASC numbers elicited after parenteral vaccination do not correlate with subsequent antibody responses [45,53]; although one detailed study found a correlation [54]. Whether this is a function of timing of measurement of antibody responses or if long lived plasma cells rather than ASCs may be a better correlate of antibody responses or whether the quality of antibody responses rather than peak titers may be a better readout remains to be understood. Investigating the somatic mutation status of the immunoglobulin heavy chain variable genes in plasmablasts would provide insight into the origin of the response as mutations progressively accumulate on variable genes after repeated immunizations [55]. Plasmablasts with a high number of somatic mutations have undergone extensive affinity maturation and selection, suggesting reactivation of memory B cells. Whilst plasmablasts with lower mutational frequencies are probably indicative of a primary response. Detailed bioinformatics analyses into the immunoglobulin gene repertoire could provide an in-depth insight into B cell dynamics driving the evolution of antibody responses.

In the tonsils, this is associated with a significant decrease in both naïve/effector (CD45RA+) and memory (CD45RO+) CD4+ cells upon vaccination [56]. Upon antigen re-encounter, memory B cells (MBC) differentiate into plasmablasts secreting IgG antibodies and undergo secondary affinity maturation to drifted influenza epitopes. Early HAI antibody responses after pandemic H1N1 vaccination provide an indicator of the long-term protective memory B cells response (CD3-CD19^+^CD20^+^CD27^+^) after a booster vaccination in low responders [53].

Interestingly, recent work investigating the plasmablast response to IIV found a lower induction of plasmablasts and plasmablast-derived polyclonal antibodies to homologous and heterologous HA proteins following IIV compared to LAIV [57]. A recent study has found a reduced number of lineages of antibody repertoire in elderly people compared to younger individuals, suggesting a reduced pool of antibody lineages for reactivation upon vaccination [58]. The use of systems biology to examine early molecular signatures after vaccination, found that expression of kinase CaMKIV at day 3 after IIV, inversely correlated with subsequent antibody titers [59].

The response of classical CD4^+^ and CD8^+^ T-cells following IIV is less clearly understood. Cross-priming by inactivated vaccines to induce MHC-Class I restricted CD8+ T-cells has been demonstrated in animal models. Data from clinical trials investigating the induction of antigen-specific CD8+ T-cells after inactivated vaccination have reported mixed results. He and colleagues reported no increase in CD8+IFNg+ T-cells after vaccination in adults or children [60,61]. However, other investigators have found an increase in CD8+IFNg+ T-cells following vaccination specific to the HA protein or live virus [62,63]. Our work has shown no increase in CD8^+^ T-cells after parenteral inactivated vaccination in adults (unpublished data). CD4+ T-cells play a key role in anti-influenza immunity both in direct killing through intrinsic effector mechanisms and by stimulating cells of the adaptive or innate immune system. In contrast to CD8+ T-cells, the influenza-specific Th1 CD4+ T-helper cell response to vaccination increases following vaccination, albeit the durability of this response is less clear. The influenza-specific CD4+ T helper (Th) 1 cell response was positively correlated with the long-term IgG MBC response, suggesting a role for this T-cell subset in mediating the long-lived MBC response [55]. More recently, work has focused on a particular subset of CD4+ T-cells, T-follicular helper cells that are critical to germinal center formation and B cell help. Two recent papers have shown that the induction of CD4+CXCR5+ TFH cells following IIV was associated with the rise in antibody response, although the phenotype of these circulating TFH cells identified to correlate with antibody induction were different [64,65].

Novel adjuvants such as proprietary oil-in-water emulsions (AS03, MF59) have shown great potential for pandemic vaccines with antigen dose-sparing and augmenting immune response to homologous and cross-reactive strains after H5 vaccination [66].

## 6. Live Attenuated Influenza Vaccine (LAIV)

**Figure 3:**
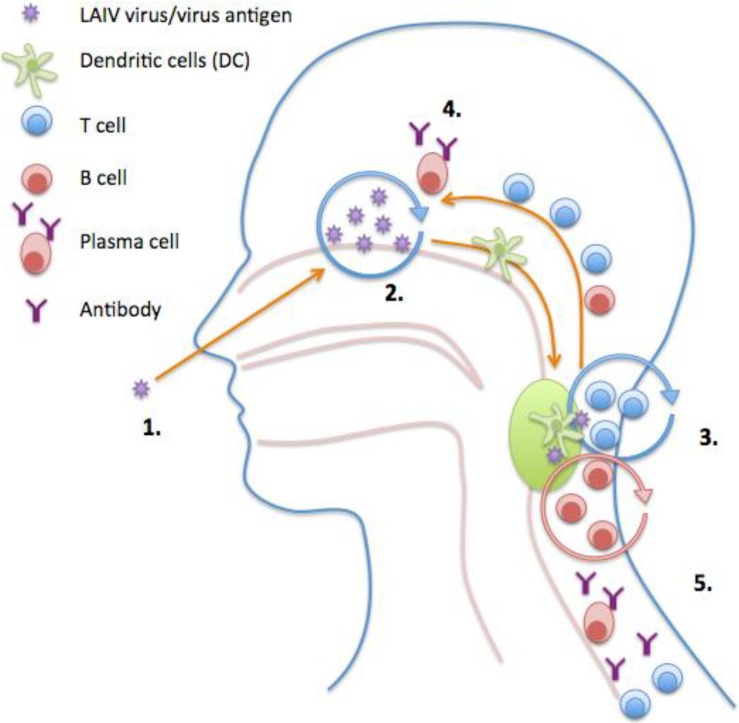
Model of induction of immune responses after live attenuated influenza vaccination (LAIV). (**1**) Intranasal LAIV immunization; (**2**) Viral antigen is transported to the tonsils/adenoids by the Dendritic Cells (DCs); (**3**) Activation and proliferation of T and B cells in tonsils/adenoids with help from CD4^+^ T-cells. Affinity maturation of B cells; (**4**,**5**) Activated T and B cells home to site of infection and enter circulation. Plasma cells secrete antibody into the blood and at the mucosal surfaces.

An alternative vaccine is the LAIV that is licensed for use in the US, Europe, Russia and India. LAIV is more efficacious in children compared to IIV with meta-analysis reporting up to 80% efficacy in children below 6 years of age to matched strains and 40% efficacy in adults [2,30]. LAIV is administered intranasally and may elicit a longer-lasting, broader immune (humoral and cellular) response, which more closely resembles natural immunity after infection (Figure 3). The use of an intranasal influenza vaccine has clear potential benefits over traditional parenteral administration of the vaccine, particularly in children e.g., long lasting effect, ease of administration, and compliance rates and provides a more appropriate immune response mimicking natural infection.

In contrast to IIV, LAIV induces a more multifaceted response with induction of serum HI, IgG, neutralizing antibody and neuraminidase antibody, local IgA and antigen-specific cytokine-secreting T-cells. LAIV must replicate in the upper respiratory tract to elicit an immune response, therefore the presence of pre-existing antibodies or cross-reactive T cells could inhibit virus infection and replication. The systemic strain-specific antibody and T-cell responses induced by LAIV in children are maintained for up to 1 year after vaccination [67]. A large field study found that the majority of subjects with high IFN-γ secreting cells (≥ 100 spot forming cells per million lymphocytes) were protected from influenza infection [68], but whether these responses were CD8+, CD4+ or NK cells was unclear. Interestingly, Hoft and colleagues demonstrated that LAIV but not IIV was able to induce CD8+ T-cells and gamma/delta T-cells in young children [69] while others have reported an increase in NK cells following influenza vaccination [62]). Mucosal antibodies are thought to be associated with protection [26,70], although a quantitative correlate does not exist in part because of the difficulty in sampling and assaying mucosal antibodies [26]. At present there are no known correlates of protection after LAIV and because of this lack of a suitable correlate of protection to aid in licensing of the LAIV, the CHMP requires animal challenge studies for annual licensing of LAIV [34]. These studies are conducted to show that LAIV significantly decreases the challenge virus in the upper respiratory tract infection as well as preventing replication of a circulating virus in the lungs. Future studies of LAIV should focus on defining the immunological mechanism of protection. The hope is that with the next generation of immunology integrated with systems biology we will gain further understanding of the mechanism of protection of LAIV.

## 7. Conclusions

The two main types of influenza vaccines induce fundamentally different immune responses and may have different mechanisms of protection (Table 3). IIVs are safe and effective against homologous vaccine and are recommended for children from 6 months old mediating protection through antibodies directed at the HA surface glycoprotein. The intranasal LAIV, recommended for children above the age of 2 years, induces a broader immune response wherein protection is not antibody mediated and probably involves undefined multiple correlates of protection. At present the two major hurdles to the widespread use of LAIV are its contraindication in some risk groups and the lack of immunological correlates of protection. The development of the next generation influenza vaccines require increased understanding of immune responses to current vaccines and infection with further evaluation of immunological correlates of protection. It is clear that the immune response and therefore correlates of protection may differ according to the vaccine type and formulation, age of the recipient and health status, and, therefore, the idea that one size fits all may not be appropriate.

**Table 3:** Comparison of the immune response to inactivated influenza and live attenuated influenza vaccine.

	Inactivated Influenza Vaccine	Live Attenuated Influenza Vaccine
HAI response	+++	+
Antibody secreting cells	++	+
Memory B cells	+	+
Nasal IgA	−/+	+++
NA antibody	−/+	++
CD4 T cells	++	+++
CD8 T cells	−	+?
Cross protective immunity	−/+	++
